# Glyphosate’s impact on vegetative growth in leafy spurge identifies molecular processes and hormone cross-talk associated with increased branching

**DOI:** 10.1186/s12864-015-1627-9

**Published:** 2015-05-19

**Authors:** Münevver Doğramacı, Michael E. Foley, David P. Horvath, Alvaro G. Hernandez, Radhika S. Khetani, Christopher J. Fields, Kathleen M. Keating, Mark A. Mikel, James V. Anderson

**Affiliations:** United States Department of Agriculture, Agricultural Research Service, Sunflower and Plant Biology Research, Fargo, ND 58102 USA; University of Illinois, W.M. Keck Center for Comparative and Functional Genomics, Urbana, IL 61801 USA; Department of Crop Sciences, 2608 Institute for Genomic Biology, and Roy J. Carver Biotechnology Center, University of Illinois, Urbana, IL 61801 USA

**Keywords:** Bud dormancy, Branching, Glyphosate, Phytohormones, Shikimate, Transcriptomics, RNAseq

## Abstract

**Background:**

Leafy spurge (*Euphorbia esula*) is a perennial weed that is considered glyphosate tolerant, which is partially attributed to escape through establishment of new vegetative shoots from an abundance of underground adventitious buds. Leafy spurge plants treated with sub-lethal concentrations of foliar-applied glyphosate produce new vegetative shoots with reduced main stem elongation and increased branching. Processes associated with the glyphosate-induced phenotype were determined by RNAseq using aerial shoots derived from crown buds of glyphosate-treated and -untreated plants. Comparison between transcript abundance and accumulation of shikimate or phytohormones (abscisic acid, auxin, cytokinins, and gibberellins) from these same samples was also done to reveal correlations.

**Results:**

Transcriptome assembly and analyses confirmed differential abundance among 12,918 transcripts (FDR ≤ 0.05) and highlighted numerous processes associated with shoot apical meristem maintenance and stem growth, which is consistent with the increased number of actively growing meristems in response to glyphosate. Foliar applied glyphosate increased shikimate abundance in crown buds prior to decapitation of aboveground shoots, which induces growth from these buds, indicating that 5-enolpyruvylshikimate 3-phosphate (EPSPS) the target site of glyphosate was inhibited. However, abundance of shikimate was similar in a subsequent generation of aerial shoots derived from crown buds of treated and untreated plants, suggesting EPSPS is no longer inhibited or abundance of shikimate initially observed in crown buds dissipated over time. Overall, auxins, gibberellins (precursors and catabolites of bioactive gibberellins), and cytokinins (precursors and bioactive cytokinins) were more abundant in the aboveground shoots derived from glyphosate-treated plants.

**Conclusion:**

Based on the overall data, we propose that the glyphosate-induced phenotype resulted from complex interactions involving shoot apical meristem maintenance, hormone biosynthesis and signaling (auxin, cytokinins, gibberellins, and strigolactones), cellular transport, and detoxification mechanisms.

**Electronic supplementary material:**

The online version of this article (doi:10.1186/s12864-015-1627-9) contains supplementary material, which is available to authorized users.

## Background

After commercialization of glyphosate [N-(phosphonomethyl)glycine] in 1974, it has become one of the most common broad spectrum herbicides used in agricultural systems [1]. However, the evolution of glyphosate-resistant weeds has increased the cost of weed management, reduced yield potential, and mandated the need for new weed management strategies [2]. No evidence of naturally occurring glyphosate-resistant plants was reported before the introduction of glyphosate-resistant transgenic crops, although several reports did indicate that biotypes of field bindweed (*Convolvulus arvensis*) and bermudagrass (*Cynodon dactylon*) were somewhat tolerant to glyphosate [3, 4]. Laticiferous perennial weeds such as hemp dogbane (*Apocynum cannabinum*), common milkweed (*Asclepias syriaca*), and leafy spurge (*Euphorbia esula*) have also been reported as glyphosate tolerant weeds [5–7]. Regardless, control of invasive perennial weeds in non-cultivated ecosystems of North America, including leafy spurge, generally requires integrated pest management programs that incorporate the use of glyphosate [8].

Reproduction of leafy spurge occurs through both seeds and vegetative propagules; however, persistence is mainly attributed to vegetative reproduction from an abundance of underground adventitious buds (UABs) [9]. Dormancy in UABs of leafy spurge also contributes to escape from conventional control measures and long-term management often requires follow up application with herbicides. Because glyphosate is a non-selective herbicide and impacts desired plants as well, in long-term management programs glyphosate is often used at lower than recommended rates or in combination with other herbicides. In fact, recommended rates of glyphosate for pastures, rangelands, roadsides, riverbanks and recreational areas (~1 kg ha^−1^) destroy the aboveground shoots of leafy spurge plants; however, it does little or no damage to UABs, and leafy spurge regenerates from these UABs. However, greater concentrations of glyphosate (~2-6 kg ha^−1^) generally cause sub-lethal effects in underground root system of leafy spurge; these higher rates still do not destroy the plants completely, but induce uncontrolled growth patterns (e.g., tillering and branching) from UABs in following generations, similar to the glyphosate-induced tillering observed in quackgrass (*Agropyron repense*) and common milkweed [10, 11]. Further, the effects of glyphosate on leafy spurge [12] were proposed to involve glyphosate’s effect on release of apical dominance in lateral buds similar to that observed in sorghum (*Sorghum bicolor*), soybean (*Glycine max*), and pea (*Pisum sativum*), which have been linked to changes in auxin levels and transport [13, 14].

Translocation of glyphosate to the apical meristems, root meristems, and underground reproductive organs of perennial plants [15] inhibits nuclear encoded and chloroplast localized [16] 5-enolpyruvylshikimate-3-phosphate synthase (EPSPS) of the shikimate biosynthetic pathway [17, 18]. Glyphosate’s known mode of action occurs by inhibiting the EPSPS-catalyzed conversion of shikimate-3-phosphate (S3P) to 5-enolpyruvylshikimate 3-phosphate (EPSP) by forming an EPSPS–S3P–glyphosate complex that competes for phosphoenolpyruvate binding [19–21]. These are critical steps for the production of chorismate, which is a precursor of aromatic amino acids (phenylalanine, tyrosine and tryptophan), auxin, and many other secondary products essential for plant growth and development. Thus, the glyphosate-induced inhibition of EPSPS reduces chorismate production leading to increased accumulation of shikimate [22]. However, beyond the known mode of action, the impact of glyphosate-treatment on transcriptional regulation has mainly focused on leaf tissues exposed to glyphosate in annual [23–25] and perennial grass species [26, 27]. Sub-lethal concentrations of foliar applied glyphosate also results in new shoot growth from UABs that produce a phenotype with reduced main stem elongation and increased branching from axillary bud outgrowth [28]. Thus, investigating glyphosate’s impact on molecular processes and phytohormones affecting vegetative regrowth from UABs could provide new insights for novel weed management solutions.

The impact of foliar applied glyphosate has already provided some insights into the involvement of molecular processes associated with auxin, ethylene, and gibberellic acid (GA) biosynthesis and signaling pathways in crown buds of leafy spurge [28]. The most significant changes observed in these UABs were for *ENT-COPALYL DIPHOSPHATE SYNTHETASE 1*, which is involved in a committed step for GA biosynthesis, and auxin transporters including *PIN-FORMED* (*PINs*), *PIN-LIKES* (*PILS*) and *ATP-BINDING CASSETTE* (*ABC*) *TRANSPORTERS*. Further, most of the significantly affected processes were associated with the endoplasmic reticulum, suggesting that this organelle plays some role in cellular physiology in response to glyphosate-treatment in leafy spurge. However, since Doğramacı et al. [28] only examined a select set of transcripts from UABs, it may not reflect the effects of foliar applied glyphosate on global gene expression or actual changes in phytohormone and shikimate levels. Thus, to gain further insights into glyphosate’s impact on molecular processes associated with phenotypes showing reduced stem elongation and increased branching due to axillary bud outgrowth, our objectives were 1) using RNAseq to determine global transcriptome abundance in aerial tissues derived from crown buds of glyphosate-treated and -untreated leafy spurge, 2) obtain shikimate and phytohormone concentration profiles from the same samples, and 3) elucidate relationships between transcriptome, shikimate, and phytohormone profiles to identify molecular processes that are altered by glyphosate.

## Results

### Vegetative growth and phenotypic changes in response to glyphosate

**Fig. 1:**
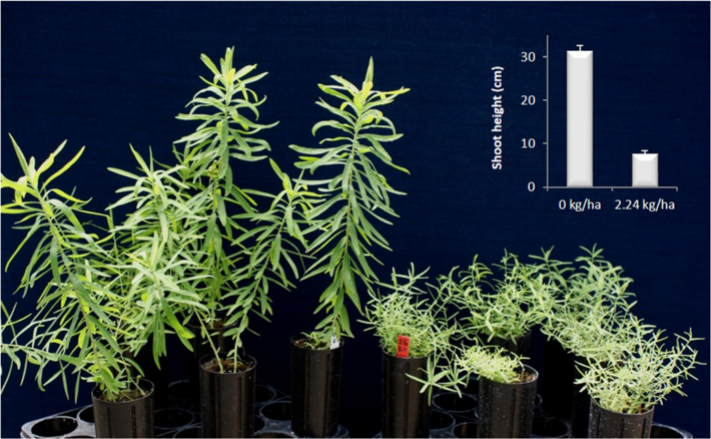
Vegetative growth from crown buds of leafy spurge post glyphosate-treatment. After foliar glyphosate-treatment i.e., 0 kg ha^−1^ as controls (left) or 2.24 kg ha^−1^ (right), plants were maintained for seven days under growth-conducive conditions and aerial tissues were decapitated to induce vegetative growth. Image and embedded chart show a significant reduction in vegetative growth six weeks post-decapitation. Vertical bars in the embedded chart indicate 95 % confidence limits

Initiation of new vegetative shoot growth from crown buds following foliar glyphosate-treatment does not occur until the directly treated aerial tissues are decapitated [28], suggesting that paradormancy in crown buds is maintained, to some extent, post-treatment by apical dominance [29, 30]. However, after decapitation of aerial tissues (7 d post-treatment), stem elongation of new vegetative shoots derived from crown buds of glyphosate-treated plants was reduced (*P* < 0.05) compared to controls (Fig. 1), and the new shoots had increased branching. This phenotype was observed in at least three subsequent shoot generations, i.e., new aerial shoots derived from UABs present on the root system of the treated plants (see Additional file 1).

### Transcriptome changes in aerial tissues

Transcriptome analyses (see Additional file 2) revealed that 12,918 transcripts had significant changes in abundance (FDR ≤ 0.05) and 6,239 of these transcripts had significant changes ≥ 2-fold in either direction; 3,632 decreased and 2,607 increased abundance in glyphosate-treated samples relative to controls. Of the 12,918 differentially expressed genes, 9,283 transcripts had similarity (BlastX hits at E values < 10E-3) to genes in the Arabidopsis database (www.arabidopsis.org). Therefore, leafy spurge transcripts discussed throughout this manuscript are based on the most homologous match to Arabidopsis (see Additional file 2). Quantitative real-time PCR (qRT-PCR) based on a select set of genes (see Additional file 3) was used to validate the reliability of the RNAseq data, which revealed a similar trend in expression patterns - with the exception of *CKX1*. Additionally, Pearson’s (P-value = 5.1E-05) or Spearman’s (P-value = 0.012) correlation coefficients confirmed significant correlation between RNAseq and qRT-PCR data and validated use of the global transcript dataset.

### Shikimate abundance and chorismate biosynthesis

**Fig. 2:**
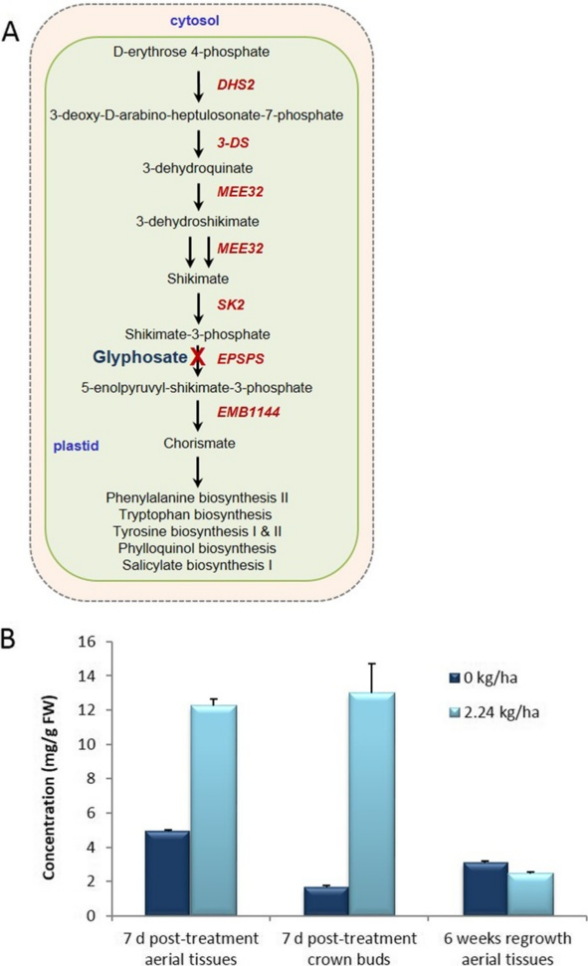
Simplified shikimate/chorismate biosynthesis pathway, and shikimate abundance in leafy spurge tissues after glyphosate-treatment. (**a**) The pathway links the metabolism of carbohydrates to the biosynthesis of the aromatic amino acids (phenylalanine, tyrosine and tryptophan). The pathway is specific to microorganisms and plants; in plants the pathway appears to occur in plastids. Glyphosate is known to inhibit 5-enolpyruvylshikimate-3-phosphate synthase (EPSPS) of the chorismate biosynthetic pathway. Red italic text indicates the genes involved in particular step/s of the pathway. (**b**) Shikimate abundance 7 days (d) post-treatment in aerial tissues which were directly exposed to glyphosate, in crown buds which were indirectly exposed to glyphosate 7 d post-treatment, and in aerial tissues derived from crown buds of foliar glyphosate-treated leafy spurge plants after decapitation (7 d post-treatment) and regrowth for six weeks. Shikimate abundance data represent the mean of four biological and two technical replicates obtained from fresh tissue (FW); vertical bars indicate ± SE of the mean

In this study, the abundance of shikimate increased in tissues directly (aerial tissue) or indirectly (crown buds) exposed to glyphosate (Fig. 2), which is likely the result of glyphosate’s inhibition of EPSPS [17, 18]. However, the abundance of shikimate in new aerial shoots (six weeks after growth-inducing decapitation) derived from crown buds of glyphosate-treated plants were similar to controls (Fig. 2). Additionally, abundance of transcripts (i.e., *EPSPS*, *EMB1144*, *SK1*) involved in various stages of chorismate biosynthesis had little change in amplitude (Table 1).

**Table 1:** Changes in abundance of transcripts involved in chorismate, auxin and strigolactone biosynthesis/signaling

Category	Gene ID Abv.	Gene ID	log2 FC	TAIR ID	Component number
Chorismate	EMB1144	EMB1144, EMBRYO DEFECTIVE 1144	0.44	AT1G48850.1	comp72334_c0
biosynthesis	EPSPS	5-ENOLPYRUVYLSHIKIMATE-3-PHOSPHATE SYNT.	0.82	AT2G45300.1	comp78915_c0
	SK1	SHIKIMATE KINASE 1	1.08	AT2G21940.5	comp71816_c0
Tryptophan	TSB2/TRP2	TRYPTOPHAN SYNTHASE BETA-SUBUNIT 2	−3.69	AT4G27070.1	comp79735_c0
biosynthesis	TSBTYPE2	TRYPTOPHAN SYNTHASE BETA TYPE 2	1.31	AT5G38530.1	comp76554_c0
Auxin	CYP79B2	CYTOCHROME P450, FAMILY 79	−4.59	AT4G39950.1	comp78594_c0
biosynthesis	CYP83B1	CYTOCHROME P450, FAMILY 83	−2.28	AT4G31500.1	comp82508_c1
	TAA1	TRYPTOPHAN AMINOTRANSFERASE ARABIDOPSIS1	4.73	AT1G70560.1	comp56536_c0
	YUC4	YUCCA 4	2.37	AT5G11320.1	comp74711_c0
	YUC9	YUCCA 9	3.12	AT1G04180.1	comp73458_c0
Auxin response	ARF2	AUXIN RESPONSE FACTOR 2	−1.16	AT5G62000.3	comp82702_c0
factors	ARF5	AUXIN RESPONSE FACTOR 5	1.86	AT1G19850.1	comp81090_c0
	ARF6	AUXIN RESPONSE FACTOR 6	1.05	AT1G30330.1	comp81172_c0
	ARF16	AUXIN RESPONSE FACTOR 16	−1.34	AT4G30080.1	comp72272_c0
	ARFP	AUXIN-RESPONSIVE FAMILY PROTEIN	−1.68	AT5G35735.1	comp76581_c1
Auxin response	IAA19	INDOLE-3-ACETIC ACID INDUCIBLE 19	2.07	AT3G15540.1	comp65987_c0
	IAA26	INDOLE-3-ACETIC ACID INDUCIBLE 26	1.49	AT3G16500.1	comp76858_c0
	IAA29	INDOLE-3-ACETIC ACID INDUCIBLE 29	−1.86	AT4G32280.1	comp68748_c0
	LRP1	LATERAL ROOT PRIMORDIUM 1	3.26	AT5G12330.4	comp80582_c0
	SAUR32	SMALL AUXIN UPREGULATED RNA 32	−1.92	AT2G46690.1	comp72184_c0
	SAUR55	SMALL AUXIN UPREGULATED RNA 55	−1.42	AT5G50760.1	comp76265_c0
	SAUR59	SMALL AUXIN UPREGULATED RNA 59	−1.34	AT3G60690.1	comp73582_c0
Auxin	LAX2	LIKE AUXIN RESISTANT 2	1.84	AT2G21050.1	comp74023_c1
transporters	LAX3	LIKE AUXIN RESISTANT 3	1.01	AT1G77690.1	comp78151_c1
	PID	PINOID	2.01	AT2G34650.1	comp81377_c0
	PIN1	PIN-FORMED 1	1.82	AT1G73590.1	comp75801_c0
	PIN1	PIN-FORMED 1	1.38	AT1G73590.1	comp80021_c0
	ABCB19	ATP-BINDING CASSETTE B19	2.39	AT3G28860.1	comp83655_c0
Auxin conjugate	IAR3	IAA-ALANINE RESISTANT 3	−1.95	AT1G51760.1	comp74098_c0
metabolism	ILL6	IAA-LEUCINE RESISTANT (ILR)-LIKE GENE 6	−1.30	AT1G44350.1	comp77344_c0
Strigolactone	MAX1	MORE AXILLARY BRANCHES 1/CYTOCHROME P450	0.64	AT2G26170.1	comp73668_c0
biosynthesis	MAX3	MORE AXILLARY BRANCHES 3	1.52	AT2G44990.1	comp68151_c0
Strigolactone sig.	MAX2	MORE AXILLARY BRANCHES 2	−0.28	AT2G42620.1	comp84166_c0

Transcript changes after foliar glyphosate treatment (2.24 kg ha^−1^) compared to controls in aerial tissues derived from crown buds of leafy spurge. The Arabidopsis Information Resource was used to annotate homologs of leafy spurge transcripts (TAIR ID), to obtain gene IDs and abbreviations (Abv.); values represent log2 fold changes (FC)

### Glyphosate-treatment impacts molecular mechanisms regulating the shoot apical meristem

Sub-network enrichment analysis (SNEA) identified numerous over-represented ontologies among transcripts with increased or decreased abundance (see Additional file 4) in aerial shoots derived from crown buds of glyphosate-treated plants. An overview of the SNEA data indicated that hubs associated with increased transcript abundance are involved with numerous processes (e.g., cell cycle, cell differentiation and proliferation, cytokinesis, DNA replication, organ formation), proteins (e.g., ASYMMETRIC LEAVES1/2, CLAVATA3, CUP-SHAPED COTYLEDON1/2, E2F TRANSCRIPTION FACTOR, KNOTTED-LIKE FROM ARABIDOPSIS THALIANA1, SHOOT MERISTEMLESS, WUSCHEL), and small molecules (i.e., brassinolide, cytokinin) associated with shoot apical meristem (SAM) maintenance. However, with the exception of the process ‘stem growth’ (see Additional file 4), most hubs associated with decreased transcript abundance are involved in stress responses (e.g., apoptosis, photoinhibition, protein degradation, ROS generation, and virulence and xenobiotic clearance).

### Phytohormone profiling and abundance of associated transcripts

Many of the aforementioned processes involved in SAM maintenance are regulated by phytohormone signaling and cross-talk [31, 32]. Indeed, previous studies [28] confirmed that pathways associated with phytohormone biosynthesis or signaling are impacted in crown buds of glyphosate-treated plants. To obtain a better understanding for the phenotypes observed in this study (Fig. 1), which could not be attributed to shikimate levels (Fig. 2) or transcripts involved in chorismate biosynthesis (Table 1), we quantified the abundance of phytohormones (see Additional file 5) in the vegetative shoots derived from crown buds of glyphosate-treated and control plants and their correlation to global transcriptome abundance.

### Auxin abundance and associated transcripts

**Fig. 3:**
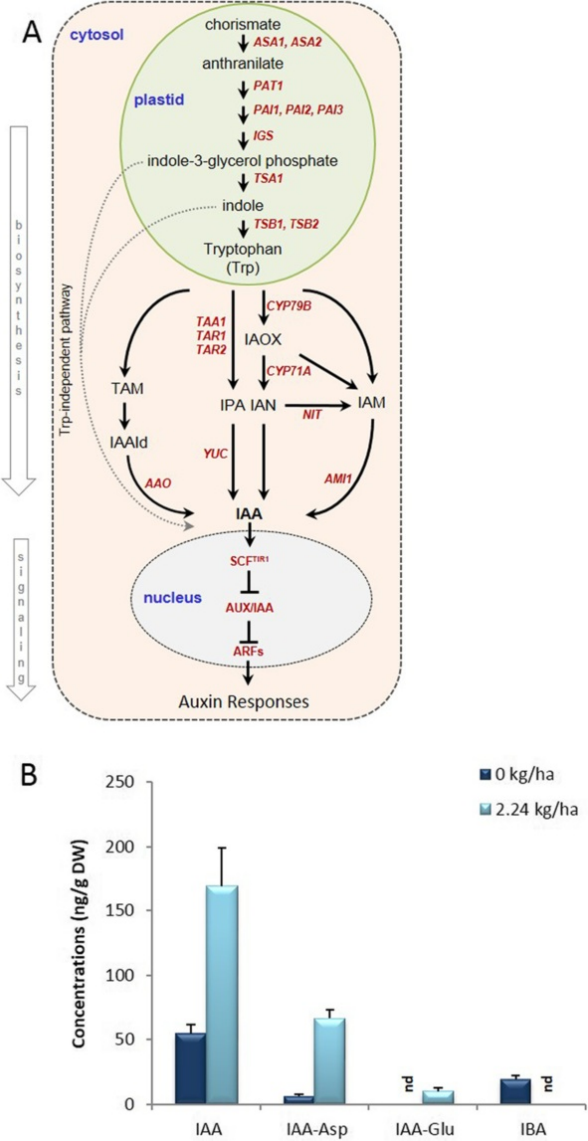
Simplified auxin biosynthesis and signaling pathway in plants, and abundance of auxin profiles in aerial tissues derived from crown buds of glyphosate-treated leafy spurge. (**a**) Two major pathways have been proposed for auxin biosynthesis in plants, i.e., tryptophan (Trp)-dependent and -independent pathways. Several Trp-dependent auxin biosynthesis pathways have been proposed, i.e. the tryptamine (TAM), the indole-3-pyruvic acid (IPA), the indole-3-acetaldoxime (IAOX), and the indole-3-acetamide (IAM) pathways. Indole-3-glycerol phosphate is the branching point of the Trp-independent route. Red italic text indicates the genes involved in particular step/s of the pathway. (**b**) Abundance of bioactive auxins (IAA, IBA) and auxin conjugates (IAA-Asp, IAA-Glu) in aerial tissues derived from crown buds of glyphosate-treated plants. Data represent the mean of four biological replicates obtained from lyophilized tissue (DW), ‘nd’ indicates ‘not detectable’; vertical bars indicate ± SE of the mean

New aerial tissues originating from crown buds of glyphosate-treated plants had an increased abundance of auxins (Fig. 3), mainly represented by biologically active indole-3-acetic acid (IAA) and smaller amounts of IAA-conjugates aspartic acid (IAA-Asp) and glutamic acid (IAA-Glu). Noteworthy, the IAA precursor indole-3-butyric acid (IBA) is present in untreated controls, but was not detectable in tissues derived from treated plants (Fig. 3). Abundance of transcripts involved in the tryptophan biosynthetic pathway (the main precursor of auxin) had varied expression patterns (Table 1). However, with the exception of transcripts homologous to Arabidopsis *TSB2* (AT4G27070, decreased abundance; Table 1) and *TSBTYPE2* (AT5G38530, increased abundance; Table 1), the majority of transcripts involved in tryptophan biosynthesis did not show significant changes in aerial tissues derived from crown buds of glyphosate-treated plants (see Additional file 2).

Tryptophan-dependent IAA biosynthesis is proposed to diverge into four pathways in plants [33, 34], which include the indole-3-acetamide (IAM), the indole-3-pyruvic acid (IPA), the tryptamine (TAM), and the indole-3-acetaldoxime (IAOX) pathways (Fig. 3). In subsequent-generations of aerial shoots obtained from crown buds of glyphosate-treated plants, transcripts homologous to Arabidopsis genes involved in the IPA pathway had increased abundance (*TAA1*, *YUC4*, *YUC9*; Table 1), whereas transcripts involved in the IAOX (*CYP79B2*, *CYP83B1*; Table 1) pathway had decreased abundance and no significant changes were observed for transcripts in the TAM and IAM pathways. Although overexpressing *CYP79B2* in Arabidopsis results in increased levels of free auxin [35], in this study, auxin levels were increased (Fig. 3) even though transcript abundance for a leafy spurge homolog of *CYP79B2* (of the IAOX pathway) was significantly decreased (−4.59 log2-fold; Table 1). Thus, the results presented in this study indicate that auxin is likely synthesized through the IPA pathway in subsequent generations of aerial tissues derived from plants exposed to glyphosate.

The increased abundance of auxin and/or its conjugates in response to glyphosate correlates well with the significant changes observed for transcripts homologous to Arabidopsis genes involved in auxin responses, which have either increased (*ARF5*, *ARF6*, *GH3.1*, *IAA19*, *IAA26*, *LRP1*) or decreased (*ARF2*, *ARF16*, *ARFP*, *IAA29*, *SAUR32*, *SAUR55*, *SAUR59*) abundance (Table 1). Other homologs of interest included several auxin transporters (*ABCB19*, *LAX2*, *LAX3*, *PID*, *PIN1*), which have a significant increase in transcript abundance. Although strigolactone abundance was not measured in this study, several homologs of transcripts involved in strigolactone biosynthesis (*MAX1* and *MAX3*) were increased; whereas, abundance of transcript involved in downstream strigolactone signaling (*MAX2*) was decreased. Strigolactones can inhibit branching by depleting auxin transporters such as PIN1 [31, 32]. Thus, assuming these transcripts perform similar function in leafy spurge, the increased abundance of a transcript homologous to Arabidopsis *PIN1* (Table 1), along with the increased branching observed in response to glyphosate treatment (Fig. 1) suggest that strigolactone biosynthesis or signaling are negatively impacted.

### Cytokinins and cell division

**Fig. 4:**
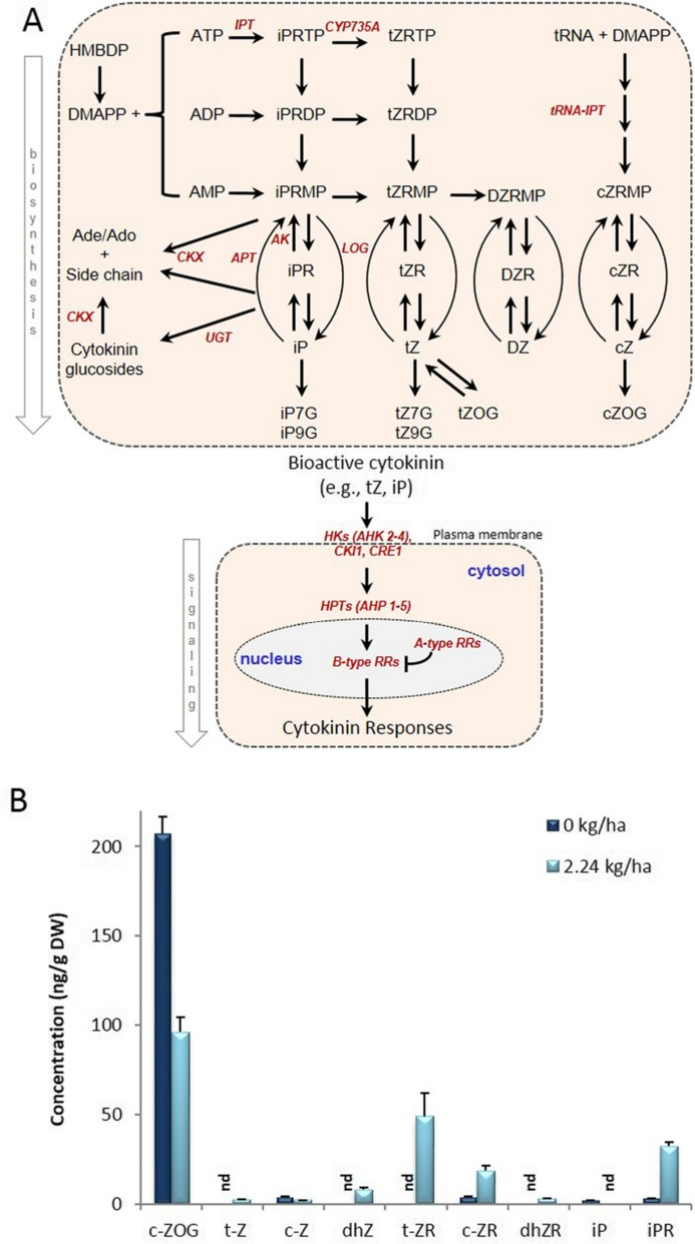
Simplified cytokinin biosynthesis and signaling pathway in plants, and abundance of cytokinin profiles in aerial tissues derived from crown buds of glyphosate-treated leafy spurge. (**a**) Adenosine phosphate-isopentenyltransferase (IPT) catalyzes the first step in the biosynthesis of isoprene cytokinins; dimethylallyl diphosphate (DMAPP) or hydroxymethylbutenyl diphosphate (HMBDP) may be used as prenyl donors, and ATP, ADP, or AMP may be used as substrates to produce diverse classes of cytokinins. In cytokinin signaling pathway, hybrid histidine protein kinases (HKs) serve as cytokinin receptors, histidine phosphotransfer proteins (HPTs) transmit the signal from HKs to nuclear response regulators (A- and B-type RRs). Red italic text indicates the genes involved in particular step/s of the pathway. (**b**) Abundance of cytokinin precursors (c-ZR, t-ZR, dZR, iPR), bioactive cytokinins (t-Z, and dhZ), and cytokinin catabolism products (c-Z, c-ZOG) in aerial tissues derived from crown buds of glyphosate-treated plants. Data represent the mean of four biological replicates obtained from lyophilized tissue (DW), ‘nd’ indicates ‘not detectable’; vertical bars indicate ± SE of the mean

Aerial shoots derived from crown buds of foliar glyphosate-treated plants were more abundant in precursors (c-ZR, t-ZR, dhZR, iPR) and bioactive cytokinins (t-Z, dhZ) than untreated controls (Fig. 4). Although larger amounts of c-ZOG (a cytokinin catabolism product of c-Z) were present in control plants, a significant level of c-ZOG was also observed in glyphosate-treated plants. Aerial shoots derived from crown buds of glyphosate-treated plants also have increased (*AK2*, *APT2*, *APT4*, *IPT3*, *LOG5*), and decreased (*LOG1*, *LOG5*, *LOG7*) abundance of transcripts homologous to Arabidopsis genes involved in cytokinin biosynthesis (Table 2), which correlates well with the increased abundance of precursor and bioactive cytokinins observed in this study (Fig. 4). Likewise, decreased abundance of transcripts involved in cytokinin metabolism (*CKX3*, *UGT73C1*, *UGT74E2*) are also consistent with the levels of bioactive and catabolized cytokinins. Transcripts responsive to cytokinins had both increased (*AHP1*, *ARR3*) or decreased (*AHK5*) abundance. Because cytokinins also play a positive role in branching [31], cell division [36], and regulation of auxin synthesis in Arabidopsis [37], the abundance of bioactive cytokinins in a subsequent generation of aerial tissue may also have some bearing on the glyphosate-induced increased branching and abundance of transcripts homologous to Arabidopsis genes involved in the cell cycle (*CDC2A*, *CDKB1*;*2*, *CYC1B*, *CYCA3*;*2*, *CYCA3*;*4*, *CYCB1*;*4*, *CYCB3*;*1*, *CYCD1*;*1*, *CYCD3*;*1*) in this study (Table 2).

**Table 2:** Changes in abundance of transcripts involved in cytokinin biosynthesis/response and cell cycle

Category	Gene ID Abv.	Gene ID	log2 FC	TAIR ID	Component number
Cytokinin	AK2	ADENOSINE KINASE 2	1.50	AT5G03300.1	comp71044_c0
biosynthesis	APT2	ADENINE PHOSPHORIBOSYL TRANSFERASE 2	4.94	AT1G80050.1	comp71670_c0
	APT4	ADENINE PHOSPHORIBOSYL TRANSFERASE 4	1.95	AT4G12440.2	comp66282_c0
	IPT3	ISOPENTENYLTRANSFERASE 3	2.04	AT3G63110.1	comp60518_c0
	LOG1	LONELY GUY 1	−1.00	AT2G28305.1	comp62804_c0
	LOG5	LONELY GUY 5	2.35	AT4G35190.1	comp70636_c0
	LOG5	LONELY GUY 5	−1.79	AT4G35190.1	comp74853_c0
	LOG7	LONELY GUY 7	−1.36	AT5G06300.1	comp62804_c1
Cytokinin	CKX3	CYTOKININ OXIDASE 3	−1.56	AT5G56970.1	comp79554_c0
metabolism	UGT73C1	UDP-GLUCOSYL TRANSFERASE 73C1	−1.01	AT2G36750.1	comp66494_c1
	UGT74E2	URIDINE DIPHOSPHATE GLYCOSYLTRANSFERASE 74E2	−1.49	AT1G05680.1	comp70147_c0
Cytokinin	AHK5	HISTIDINE KINASE 5	−1.82	AT5G10720.1	comp78257_c0
response	AHP1	HISTIDINE-CONTAINING PHOSPHOTRANSMITTER 1	1.87	AT3G21510.1	comp68321_c1
	ARR3	ARABIDOPSIS RESPONSE REGULATOR 3	2.35	AT1G59940.1	comp69766_c0
Cell cycle	CDC2A	CELL DIVISION CONTROL 2	1.36	AT3G48750.1	comp73867_c0
	CDKB1;2	CYCLIN-DEPENDENT KINASE B1;2	2.65	AT2G38620.2	comp68143_c0
	CDKB2;2	CYCLIN-DEPENDENT KINASE B2;2	2.49	AT1G20930.1	comp69392_c0
	CYC1B	CYCLIN B 1;2	2.65	AT5G06150.1	comp73117_c0
	CYCA3;2	CYCLIN-DEPENDENT PROTEIN KINASE 3;2	2.45	AT1G47210.2	comp62278_c0
	CYCA3;4	CYCLIN A3;4	2.63	AT1G47230.1	comp80977_c0
	CYCB1;4	CYCLIN B1;4	2.55	AT2G26760.1	comp66390_c0
	CYCB3;1	CYCLIN B3;1	2.22	AT1G16330.1	comp77441_c0
	CYCD1;1	CYCLIN D1;1	1.24	AT1G70210.1	comp79632_c0
	CYCD3;1	CYCLIN D3;1	1.54	AT4G34160.1	comp70052_c0
	CYCD3;2	CYCLIN D3;2	1.46	AT5G67260.1	comp68903_c0

Transcript changes after foliar glyphosate treatment (2.24 kg ha^−1^) compared to controls in aerial tissues derived from crown buds of leafy spurge. The Arabidopsis Information Resource was used to annotate homologs of leafy spurge transcripts (TAIR ID), to obtain gene IDs and abbreviations (Abv.); values represent log2 fold changes (FC)

### GA abundance and associated transcripts

**Fig. 5:**
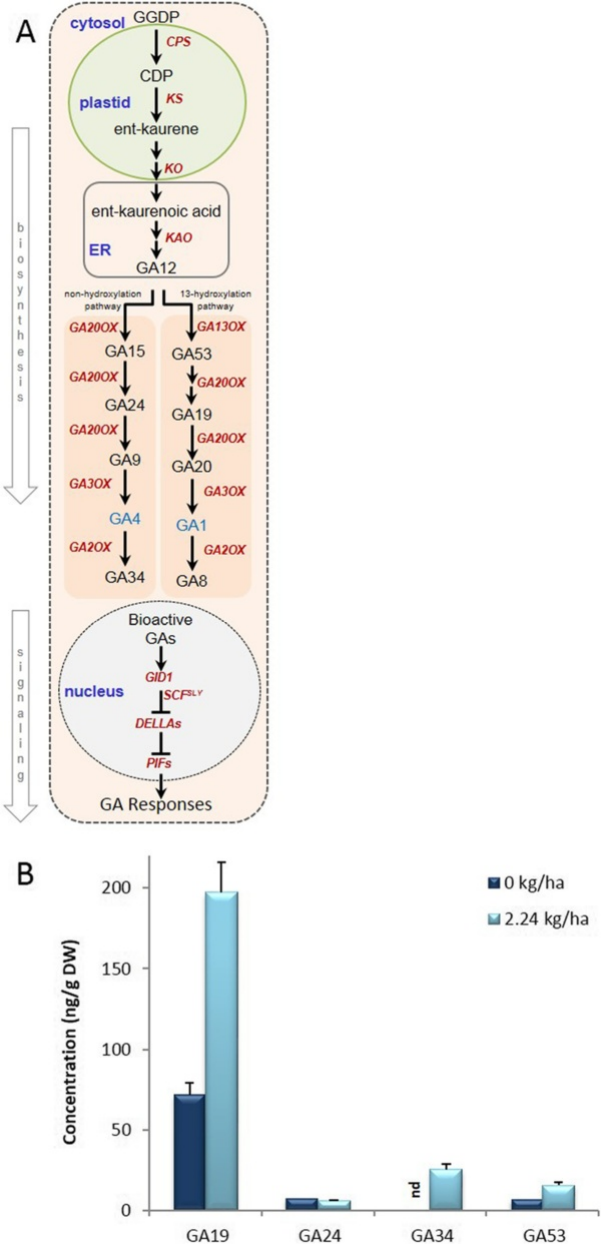
Simplified gibberellic acid (GA) biosynthesis and signaling pathway in plants, and abundance of GA profiles in aerial tissues derived from crown buds of glyphosate-treated leafy spurge. (**a**) GA biosynthesis occurs in three stages in different subcellular compartments: cyclization of geranylgeranyl diphosphate by copalyl diphosphate synthase (CPS) and ent-kaurene synthase (KS) to produce ent-kaurene in the plastids; conversion of ent-kaurene into GA12 via cytochrome P450 monooxygenases (e.g., KO, KAO) in the endoplasmic reticulum (ER); conversion of GA12 to bioactive forms of GA (e.g., GA1 and GA4 indicated with blue text) in the cytosol via 13-hydroxylation pathway or non-hydroxylation pathway. Red italic text indicates the genes involved in particular step/s of the pathway. (**b**) Abundance of GA precursors (GA19, GA24, GA53) and catabolism products (GA34). Data represent the mean of four biological replicates obtained from lyophilized tissue (DW), ‘nd’ indicates ‘not detectable’; vertical bars indicate ± SE of the mean

Total GAs (precursors and catabolites of bioactive GA) were more abundant in aerial shoots derived from crown buds of glyphosate-treated plants (Fig. 5). The GAs detected (i.e., GA19, GA24, GA34, GA53) belong to either the early 13-hydroxylation pathway or to the non-hydroxylation pathway. The increased 13-hydroxylation pathway products G19 and G53 (precursors of biologically active GA1) suggests that some components of the GA biosynthetic pathway are stimulated in subsequent generations of aerial tissues from plants exposed to glyphosate. However, the increased abundance of GA34 (catabolism product of GA4) indicates that bioactive GA4 must have been produced in aerial tissues but is rapidly catabolized. The increased abundance of GA precursors in response to glyphosate seems consistent with the increased abundance of transcripts homologous to Arabidopsis genes (*KO*, *KS*) involved in early steps of GA biosynthesis. However, the decreased abundance of a transcript homologous to Arabidopsis *GA3OX1* (Table 3) might suggest that downstream biosynthetic steps involved in the conversion of precursors to bioactive GA are inhibited in response to glyphosate. The increased abundance of transcripts (*GA2OX2*, *GA2OX4*) involved in catabolism of bioactive GA would also correlate well with the lack of bioactive GAs observed in response to glyphosate in this study. Further, transcripts of homologs known to inhibit downstream GA-signaling [38], such as the GRAS family (*SCL28*, *SCL32*) of transcription factors or the related Scarecrow family of transcription factors involved in asymmetric cell division (*SCR*, *SHR*), have increased abundance in response to glyphosate (Table 3).

**Table 3:** Changes in abundance of transcripts involved in GA biosynthesis, catabolism and signaling

Category	Gene ID Abv.	Gene ID	log2 FC	TAIR ID	Component number
GA biosynthesis	GA3OX1	GIBBERELLIN 3-OXIDASE 1	−2.73	AT1G15550.1	comp57360_c0
	KO/CYP701A3	ENT-KAURENE OXIDASE 1	1.09	AT5G25900.1	comp77748_c0
	KS	ENT-KAURENE SYNTHASE	1.38	AT1G79460.1	comp72406_c0
GA catabolism	GA2OX1	GIBBERELLIN 2-OXIDASE 1	−1.38	AT1G78440.1	comp70621_c1
	GA2OX2	GIBBERELLIN 2-OXIDASE 2	2.07	AT1G30040.1	comp72361_c0
	GA2OX4	GIBBERELLIN 2-OXIDASE 4	3.20	AT1G02400.1	comp67827_c0
	GA2OX4	GIBBERELLIN 2-OXIDASE 4	1.33	AT1G02400.1	comp76771_c0
GA mediated	GASA6	GA-STIMULATED ARABIDOPSIS 6	3.50	AT1G74670.1	comp69402_c0
signaling	GASA9	GA-STIMULATED ARABIDOPSIS 9	2.10	AT1G22690.3	comp64133_c0
	GASA11	GA-STIMULATED ARABIDOPSIS 11	−1.02	AT2G18420.1	comp74978_c0
	SCL14	SCARECROW-LIKE 14	−1.52	AT1G07530.1	comp81394_c0
	SCL28	SCARECROW-LIKE 28	2.11	AT1G63100.1	comp83623_c0
	SCL32	SCARECROW-LIKE 32	1.18	AT3G49950.1	comp65814_c0
	SCR	SCARECROW	1.41	AT3G54220.1	comp80927_c1
	SHR	SHORT ROOT	1.85	AT4G37650.1	comp70203_c1

Transcript changes after foliar glyphosate treatment (2.24 kg ha^−1^) compared to controls in aerial tissues derived from crown buds of leafy spurge. The Arabidopsis Information Resource was used to annotate homologs of leafy spurge transcripts (TAIR ID), to obtain gene IDs and abbreviations (Abv.); values represent log2 fold changes (FC)

### ABA abundance and associated transcripts

**Figure 6:**
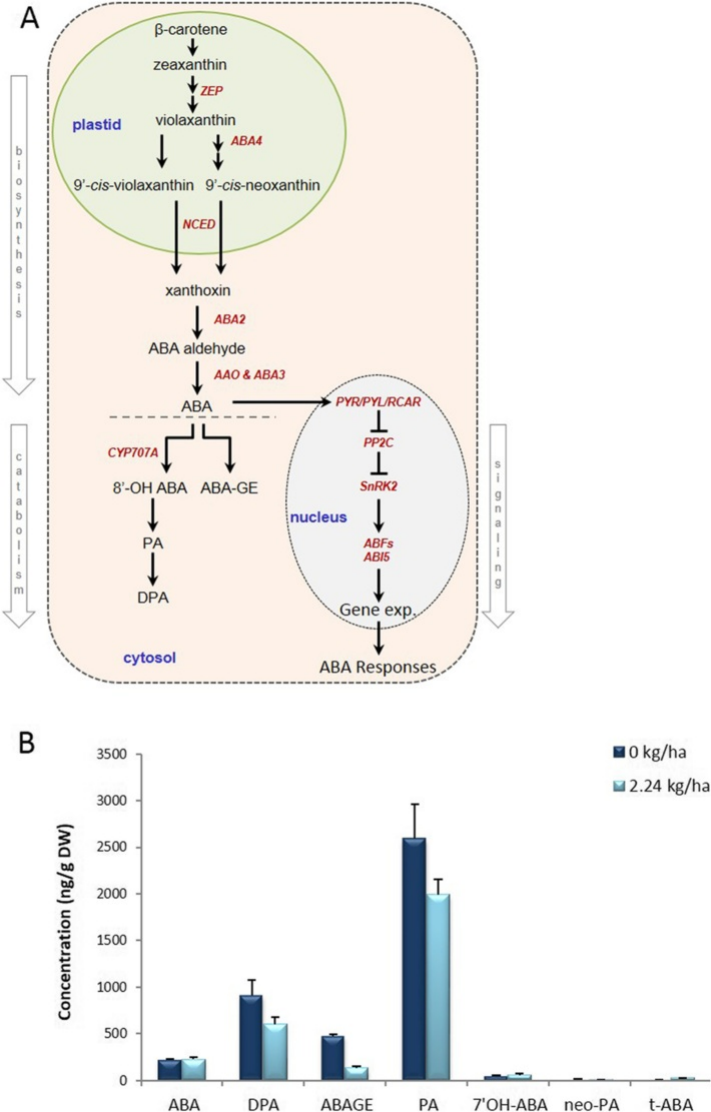
Simplified pathway of abscisic acid (ABA) biosynthesis and signaling in plants, and abundance of ABA profiles in aerial tissues derived from crown buds of glyphosate-treated leafy spurge. (**a**) ABA is synthesized from C40 carotenoids (e.g., β-carotene) via the oxidative cleavage to produce xanthoxin in the plastids; xanthoxin is converted to ABA-aldehyde and then to ABA in the cytoplasm. Red italic text indicates the genes involved in particular step/s of the pathway. (**b**) Abundance of ABA, and its metabolites and conjugates (PA, DPA, ABAGE, 7’OH-ABA, neo-PA, t-ABA). Data represent the mean of four biological replicates obtained from lyophilized tissue (DW); vertical bars indicate ± SE of the mean

Bioactive ABA levels were similar in subsequent generations of aerial tissues derived from crown buds of foliar glyphosate-treated and control plants (Fig. 6). The only significant changes observed in response to glyphosate occurred for the main ABA metabolism pathway through 8’-hydroxylation (Fig. 6), which leads to inactive phasic acid (PA) production and its conversion to diphaseic acid (DPA), and the secondary catabolism pathway leading to conjugation of ABA (ABAGE). Some transcripts homologous to Arabidopsis genes involved in ABA biosynthesis had both increased (*ABA2*, *NCED3*) and decreased (*AAO4*) abundance (Table 4) but did not seem to impact the abundance of bioactive ABA observed in treated or control plants. However, the decrease in abundance of transcripts involved in ABA catabolism (e.g., *CYP707A3*, *UGT71B1*) would be consistent with the decreased abundance of the ABA catabolites DPA and PA in glyphosate-treated plants. Transcripts involved in or responsive to ABA signaling in other systems had both increased (*COR6.6*, *DREB1D*, *PLDALPHA1*, *RCAR10*) and decreased (*ABF1*, *AP2C1*, *CDPK1*) transcript abundance. However, because changes in transcript abundance do not correlate to bioactive ABA levels, it is possible that some or all of these transcripts may be responsive to other phytohormones or stress-responses (see Additional file 2).

**Table 4:** Changes in abundance of transcripts involved in ABA biosynthesis, catabolism and signaling

Category	Gene ID Abv.	Gene ID	log2 FC	TAIR ID	Component number
ABA	AAO4	ALDEHYDE OXIDASE 4	−1.31	AT1G04580.1	comp67916_c0
biosynthesis	ABA2	ABA DEFICIENT 2	1.92	AT1G52340.1	comp64618_c0
	ABA2	ABA DEFICIENT 2	−1.06	AT1G52340.1	comp67801_c0
	ABA2	ABA DEFICIENT 2	4.64	AT1G52340.1	comp69765_c0
	NCED3	NINE-CIS-EPOXYCAROTENOID DIOXYGENASE 3	2.27	AT3G14440.1	comp62453_c0
ABA	CYP707A3	CYTOCHROME P450, FAMILY 707	−5.55	AT5G45340.1	comp48367_c0
catabolism	UGT71B1	UDP-GLUCOSYL TRANSFERASE 71B1	−1.35	AT3G21750.1	comp63205_c0
	UGT71B1	UDP-GLUCOSYL TRANSFERASE 71B1	−1.53	AT3G21750.1	comp78910_c1
ABA	ABF1	ABSCISIC ACID RESPONSIVE ELEMENT-BINDING FAC 1	−2.19	AT1G49720.2	comp81822_c0
signaling	AP2C1	PP2C-TYPE PHOSPHATASE	−1.58	AT2G30020.1	comp71725_c1
	CDPK1A	CALCIUM-DEPENDENT PROTEIN KINASE 1A	−1.21	AT1G74740.1	comp82371_c2
	COR6.6	COLD-RESPONSIVE 6.6	2.52	AT5G15970.1	comp63310_c0
	DREB1D	DEHYDRATION-RESPONSIVE ELEMENT-BINDING 1D	1.30	AT5G51990.1	comp72894_c0
	HB6	HOMEOBOX PROTEIN 6	1.07	AT2G22430.1	comp68807_c1
	PLDALPHA1	PHOSPHOLIPASE D ALPHA 1	1.37	AT3G15730.1	comp79335_c0
	RCAR10	REGULATORY COMPONENTS OF ABA RECEPTOR 10	2.37	AT2G38310.1	comp66032_c0

Transcript changes after foliar glyphosate treatment (2.24 kg ha^−1^) compared to controls in aerial tissues derived from crown buds of leafy spurge. The Arabidopsis Information Resource was used to annotate homologs of leafy spurge transcripts (TAIR ID), to obtain gene IDs and abbreviations (Abv.); values represent log2 fold changes (FC)

### Other phytohormones and growth related processes

Although abundance of ethylene and jasmonic acid (JA) were not quantified in this study, significant changes in abundance of transcripts homologous to Arabidopsis genes involved in biosynthesis or signaling associated with these phytohormones were observed (Table 5). For example, transcripts involved in JA biosynthesis/signaling (*JAR1*, *LOX2*, *WRKY40*, *WRKY50*, *WRKY70*) mainly had decreased abundance (Table 5) but a significant increase in abundance of *ST2A* and *CYP94B1* was observed in response to glyphosate. *ST2A* encodes a hydroxyjasmonate sulfotransferase in Arabidopsis, which inactivates the function of JA [39]. Additionally, transcripts involved in ethylene biosynthesis (see Additional file 6) had decreased (*ACS1*, *ACS6*, *ACS10*) and increased (*ACS11*) abundance (Table 5) in subsequent generations of aerial tissue derived from crown buds of glyphosate-treated plants. These aerial tissues also had mixed transcript abundance for ethylene responsive genes; *ERF12*, *ERF13*, *RAP2.3*, *T16I18.10*, and *TINY2* had increased transcript abundance, whereas *ERF1*, *ERF6*, *ERF105*, *ERF110*, and *K22G18.1* had decreased transcript abundance (Table 5). Because exogenous application of ethylene precursor causes a dwarfed phenotype in subsequent generations derived from UABs of leafy spurge [40], the significant changes in ethylene-responsive transcripts suggest that glyphosate likely affected ethylene biosynthesis and/or signaling pathways in UABs of leafy spurge, which could have some effect on the stunted phenotype observed in this study.

**Table 5:** Changes in abundance of transcripts involved in ethylene and JA biosynthesis, metabolism and signaling

Category	Gene ID Abv.	Gene ID	log2 FC	TAIR ID	Component number
Ethylene	ACS1	ACC SYNTHASE 1	−2.48	AT3G61510.1	comp63647_c0
biosynthesis	ACS6	ACC SYNTHASE 6	−1.60	AT4G11280.1	comp81533_c0
	ACS10	ACC SYNTHASE 10	−1.94	AT1G62960.1	comp83189_c0
	ACS11	ACC SYNTHASE 11	5.97	AT4G08040.1	comp68485_c0
AP2/ERF TF	ERF1	ETHYLENE RESPONSE FACTOR 1	−3.35	AT3G23240.1	comp63489_c0
	ERF6	ETHYLENE RESPONSIVE ELEMENT BINDING FAC 6	−1.22	AT4G17490.1	comp76450_c0
	ERF12	ERF DOMAIN PROTEIN 12	1.63	AT1G28360.1	comp69972_c0
	ERF13	ETHYLENE-RESPONSIVE ELEMENT BINDING FAC 13	3.33	AT2G44840.1	comp68474_c0
	ERF105	ETHYLENE-RESPONSIVE ELEMENT BINDING FAC 105	−2.05	AT5G51190.1	comp81745_c0
	ERF110	ETHYLENE RESPONSE FACTOR 110	−1.78	AT5G50080.1	comp78763_c0
	K22G18.1	K22G18.1	−1.94	AT5G61890.1	comp52489_c0
	RAP2.3	RELATED TO AP2 3	1.40	AT3G16770.1	comp58768_c0
	T16I18.10	T16I18.10	1.29	AT4G32800.1	comp68841_c0
	TINY2	TINY2	1.95	AT5G11590.1	comp68230_c0
JA biosynthesis	AOS	ALLENE OXIDE SYNTHASE	1.11	AT5G42650.1	comp61253_c0
	LOX2	LIPOXYGENASE 2	−2.50	AT3G45140.1	comp77454_c0
	LOX2	LIPOXYGENASE 2	−2.09	AT3G45140.1	comp79859_c0
	LOX2	LIPOXYGENASE 2	−2.11	AT3G45140.1	comp82248_c0
JA mediated	CYP94B1	CYTOCHROME P450, FAMILY 94	1.62	AT5G63450.1	comp75229_c0
signaling	JAR1	JASMONATE RESISTANT 1	−1.26	AT2G46370.2	comp80290_c0
	WRKY40	WRKY DNA-BINDING PROTEIN 40	−1.94	AT1G80840.1	comp75129_c0
	WRKY50	WRKY DNA-BINDING PROTEIN 50	−2.96	AT5G26170.1	comp62445_c0
	WRKY70	WRKY DNA-BINDING PROTEIN 70	−1.39	AT3G56400.1	comp75789_c1
	ST2A	ARABIDOPSIS THALIANA SULFOTRANSFERASE 2A	6.54	AT5G07010.1	comp85207_c0

Transcript changes after foliar glyphosate treatment (2.24 kg ha^−1^) compared to controls in aerial tissues derived from crown buds of leafy spurge. The Arabidopsis Information Resource was used to annotate homologs of leafy spurge transcripts (TAIR ID), to obtain gene IDs and abbreviations (Abv.); values represent log2 fold changes (FC)

Some *CYTOCHROME P450 MONOOXYGENASES* (*P450s*) and *UDP*-*GLUCOSYL*/-*GLYCOSYL TRANSFERASES* (*UGTs*) are classified under phytohormone biosynthesis or signaling (Tables 1–5, and Additional file 2). However, numerous other members of these two families represent the largest number of differentially expressed transcripts (see Additional file 2) in subsequent generations of aerial tissues in response to glyphosate. Similarly, numerous *ABC TRANSPORTERS* and *GLUTATHIONE S*-*TRANSFERASES* (*GSTs*) had significant changes in transcript abundance with both increased and decreased patterns (Table 6, and Additional file 2).

**Table 6:** Changes in transcript abundance of ABC Transporters and Glutathione Transferases (GSTs)

Category	Gene ID Abv.	Gene ID	log2 FC	TAIR ID	Component number
ABC	ABCG2	ATP-BINDING CASSETTE G2	1.70	AT2G37360.1	comp66100_c0
transporters	ABCG5	ATP-BINDING CASSETTE G5	1.53	AT2G13610.1	comp76645_c0
	ABCG6	ATP-BINDING CASSETTE G6	−4.14	AT5G13580.1	comp52290_c0
	ABCG6	ATP-BINDING CASSETTE G6	1.91	AT5G13580.1	comp60677_c0
	ABCG8	ATP-BINDING CASSETTE G8	1.85	AT5G52860.1	comp69526_c0
	ABCG14	ATP-BINDING CASSETTE G14	1.71	AT1G31770.1	comp75586_c0
	ABCG25	ATP-BINDING CASSETTE G25	1.12	AT1G71960.1	comp78609_c0
	ABCG40	ATP-BINDING CASSETTE G40	−2.65	AT1G15520.1	comp29716_c0
	ABCG40	ATP-BINDING CASSETTE G40	2.67	AT1G15520.1	comp60991_c0
	ABCG40	ATP-BINDING CASSETTE G40	−3.62	AT1G15520.1	comp66678_c0
	ABCG40	ATP-BINDING CASSETTE G40	−4.54	AT1G15520.1	comp66678_c1
	ABCG40	ATP-BINDING CASSETTE G40	−4.01	AT1G15520.1	comp66678_c2
	ABCG40	ATP-BINDING CASSETTE G40	−4.39	AT1G15520.1	comp66678_c3
	ABCG40	ATP-BINDING CASSETTE G40	−2.33	AT1G15520.1	comp77719_c0
GSTs	GST30	GLUTATHIONE S-TRANSFERASE 30	0.81	AT1G10370.1	comp68719_c0
	GSTF11	GLUTATHIONE S-TRANSFERASE F11	4.22	AT3G03190.1	comp70895_c0
	GSTL2	GLUTATHIONE TRANSFERASE LAMBDA 2	0.59	AT3G55040.1	comp73284_c0
	GSTU6	GLUTATHIONE S-TRANSFERASE TAU 6	−0.49	AT2G29440.1	comp79342_c1
	GSTU25	GLUTATHIONE S-TRANSFERASE TAU 25	1.48	AT1G17180.1	comp62755_c0
	GSTU25	GLUTATHIONE S-TRANSFERASE TAU 25	−0.72	AT1G17180.1	comp78043_c0
	GSTU25	GLUTATHIONE S-TRANSFERASE TAU 25	2.16	AT1G17180.1	comp84357_c0
	GSTT1	GLUTATHIONE S-TRANSFERASE THETA 1	−0.44	AT5G41210.1	comp69253_c0
	GSTZ1	GLUTATHIONE S-TRANSFERASE ZETA 1	1.03	AT2G02390.1	comp77147_c0

Transcript changes after foliar glyphosate treatment (2.24 kg ha^−1^) compared to controls in aerial tissues derived from crown buds of leafy spurge. The Arabidopsis Information Resource was used to annotate homologs of leafy spurge transcripts (TAIR ID), to obtain gene IDs and abbreviations (Abv.); values represent log2 fold changes (FC)

## Discussion

Direct exposure of leafy spurge aerial tissues to sub-lethal concentrations of glyphosate causes an indirect effect that impacts molecular processes in UABs prior to growth induction by decapitation [28]. In this study, reduced main stem elongation and increased branching was observed in four subsequent generations derived from UABs present in the same root system of treated plants (Fig. 1, and Additional file 1), and the effects of glyphosate on phenotype did not diminish in these generations. However, after four generations of vegetative growth no more UABs were left in the root system of these plants (grown in cone-tainers) to observe if the plants would ultimately outgrow the effects of glyphosate. Parallel studies performed under field conditions revealed that higher rates of foliar-applied glyphosate (3 or 6 kg ha^−1^) affects vegetative growth from UABs of leafy spurge and causes similar phenotypical alterations for at least two consecutive years after the glyphosate-treatment (Doğramaci et al. unpublished data). Sub-lethal rates of glyphosate have also been reported to induce similar phenotypes in annual species such as corn (*Zea mays*), cotton (*Gossypium hirsutum*), sorghum, and wheat (*Triticum aestivum*) [13, 41–43]. To gain insights on factors involved in these glyphosate-induced phenotypes, here we determined the effects of foliar applied glyphosate on the abundance of metabolites (shikimate and hormones) and their correlation to global transcript abundance in subsequent generation of aerial tissues.

### Glyphosate-treatment caused differential accumulation of shikimate

Little information is available on the abundance of shikimate in underground buds or in subsequent generations derived from glyphosate-treated perennials, with the exception of glyphosate-induced accumulation of shikimate in sprouted tubers of the perennial weed purple nutsedge (*Cyperus rotundus*) [44]. Overall, changes in shikimate abundance in leafy spurge tissues after glyphosate-treatment (Fig. 2) were relatively low compared to those reported in other species [45, 46], but it is important to note that the rate used in this study had only a sub-lethal effect. Minor changes in abundance of transcripts involved in the chorismate biosynthetic pathway in aerial tissues (Table 1) are similar to that previously observed in crown buds of leafy spurge [28] or other weedy species such as palmer amaranth and horseweed following glyphosate-treatment [46, 47]. The increased accumulation of shikimate in aerial tissues of leafy spurge directly treated with glyphosate or in crown buds that were indirectly exposed (Fig. 2) is not surprising, given that significant shikimate accumulation is detectable several days prior to visual injury in response to glyphosate-treatment in other species [48]. Regardless of their seasonal dormancy phase (i.e., para-, endo-, or eco-dormancy), UABs of leafy spurge are active and rapidly respond to environmental changes; in fact, during the shifts between seasonal phases there is some limited growth and development [49] suggesting UABs may serve as a sink tissue. We hypothesize that the increased abundance of shikimate in crown buds is due to either 1) translocation of glyphosate from foliar tissues into the root system and UABs to locally inhibit EPSPS, and/or 2) translocation and compartmentalization of foliar shikimate into the root system or UABs. However, there is more support for the first option based on previous reports indicating that glyphosate or its metabolites are translocated into the roots and UABs of leafy spurge [12], similar to that reported in other weedy species [15, 50].

Although crown buds of foliar glyphosate-treated leafy spurge initially had increased shikimate abundance prior to decapitation, in subsequent generations of aerial shoots (six weeks post-decapitation) shikimate abundance decreased and was similar to that observed in untreated controls (Fig. 2). These results suggest that duration of time after growth induction either allowed for dissipation of shikimate originally observed in crown buds, or EPSPS is no longer inhibited. However, the reduction in shikimate levels did not reverse the glyphosate-induced phenotype (Fig. 1, and Additional file 1). Studies in Arabidopsis revealed that shikimate can be consumed as a secondary metabolite as an ester [51]. Further, silencing of a gene (*HYDROXYCINNAMOYL*-*COA SHIKIMATE*/*QUINATE HYDROXYCINNAMOYLTRANSFERASE*) downstream of the chorismate pathway (i.e., phenylpropanoid metabolism) that involves shikimate recycling causes stunting [51] similar to that observed in this study, which appears to inhibit polar auxin transport [52]. The results of this study (Fig. 2) are consistent with abundance of shikimate initially increasing after glyphosate-treatment in other plant systems and then decreasing over time [48, 53-55]. For example, abundance of shikimate generally peaks between 4 or 7 days after glyphosate-treatment in crops such as corn, soybean (*Glycine max*), sunflower (*Helianthus annuus*), and wheat [48, 53], and in several weedy species [54].

Even though glyphosate is known to be metabolized in plants and presence of glyphosate oxidoreductase-like enzymes (involved in the degradation pathway of glyphosate in soil) have been speculated, to date no corresponding enzymes or genes have been identified [55, 56]. Therefore, we are not able to determine the extent of glyphosate metabolism based on transcript abundance in leafy spurge. Previous studies [50] have shown that ^14^C-glyphosate levels remain similar in leaves and roots of field bindweed, Canada thistle (*Cirsium arvense*), and tall morning glory (*Ipomoea purpurea*) 3–4 weeks post-application, with only a limited accumulation of the main glyphosate metabolite aminomethylphosphonic acid (AMPA). However, in glyphosate-resistant soybean, the highest levels of glyphosate and AMPA were found in treated leaves within 1 day after glyphosate treatment and gradually decreased within 3 weeks [56]. Further, while exogenous application of AMPA did not affect shikimate levels it had phytotoxic effects and induced some injuries (e.g., reduced chlorophyll content, shoot fresh weight) in glyphosate resistant and non-resistant soybean [56]; but, stunted or bushy phenotypes were not reported after AMPA application.

Because growth-induction of leafy spurge crown buds results in decreased abundance of starch and sucrose and increased abundance of fructose [57], it appears that storage reserves in the root system are remobilized into free sugars to provide an energy source for new vegetative shoot growth. It is plausible that decapitation-induced growth causes a similar process to occur where root sequestered glyphosate or its metabolites, if any, are remobilized into UABs. Remobilization of these substances could cause subsequent-generations of vegetative growth to continually be exposed to residual toxicity and impact SAM maintenance and axillary bud outgrowth. Glyphosate’s effects on meristematic tissues over extended periods in white birch (*Betula papyrifera*) has also been speculated to involve sugar flow into the root system in the fall and a reverse flow of sugar to developing leaves in the spring [58]. This hypothesis is consistent with glyphosate being mobilized to sink tissues such as growing meristems via the phloem [59]. Alternatively, it is possible that glyphosate has an irreversible effect on the main SAM of UABs prior to decapitation-induced growth. The effects of glyphosate on a select set of molecular processes in crown buds of foliar glyphosate-treated plants [28], and increased abundance of shikimate in crown buds 7 days post-treatment (Fig. 2), would support this hypothesis.

### Auxin, cytokinin, strigolactone cross-talk and shoot branching

Shoot branching is known to involve the key hormones auxin, cytokinin, and strigolactones [31, 60]. The increased abundance of transcripts homologous to Arabidopsis genes involved in the IPA biosynthetic pathway leading to auxin (Fig. 3), along with the increased levels of auxin in subsequent generations of aerial tissues from glyphosate-treated plants suggest that tryptophan-dependent auxin biosynthesis is not inhibited at this stage of development. These results also fit well with the abundance of shikimate observed in the same aerial tissues (Fig. 2). Because increased abundance of shikimate is a marker for inhibition of EPSPS, the increased abundance of auxin and similar levels of shikimate in both glyphosate treated and control plants suggest that EPSPS is not inhibited in subsequent generations of aerial shoots (Fig. 2). These results may suggest either lack of glyphosate or inhibition of its transport into plastids in these shoots, therefore not affecting the chorismate pathway or its downstream auxin biosynthesis pathway. However, we cannot rule out the possibility that the increase in auxin could also be due to an increase in the number of growing meristems.

In this study, the bushy phenotypes derived from crown buds of foliar glyphosate-treated plants do not appear to have a dominant/leading apical meristem to suppress outgrowth of axillary buds (Fig. 1), suggesting that apical dominance is lacking in the system and/or the polar auxin transport stream is affected. Previous studies have shown that exogenous application of auxin represses stem elongation of shoots derived from crown buds of leafy spurge [57, 61], which may have some bearing on the reduced main stem elongation and increased branching in response to glyphosate (Fig. 1). Indeed, auxin has been proposed to regulate shoot branching through at least two processes [31, 32]. First, the canalization theory involves the effects of apical dominance on abundance and transport of auxin in stems, which is proposed to inhibit auxin transport from axillary buds to repress axillary bud outgrowth. The second proposed mechanism involves auxin’s impact on acropetal movement of cytokinins and strigolactone into the buds to regulate bud outgrowth [62–66]. However, whereas cytokinins induce bud outgrowth, strigolactone signaling can affect PIN1 polarization in the plasma membrane to inhibit auxin transport [32].

PINs encode auxin efflux carriers that mediate tissue-specific, cell-to-cell polar auxin transport [67] critical for developmental patterning and differential growth responses [68]. Strigolactone biosynthesis or signaling mutants also have increased axillary bud outgrowth, resulting in a bushy and dwarfed phenotype in Arabidopsis [69]; increased branching is also correlated with high levels of PIN1 and auxin in the main stem [70, 71]. However, in this study, the increased transcript abundance for homologs of Arabidopsis *MAX1* and *MAX3* (Table 1), involved in the strigolactone biosynthesis [69], do not correlate well with the glyphosate-induced branching phenotype. Indeed, the minimal decrease in transcript abundance for a homolog of *MAX2*, involved in downstream strigolactone signaling through regulation of PIN1 [32] could account for the increased transcript abundance for *PIN1* (Table 1). Assuming that the leafy spurge homologs perform similar functions as their Arabidopsis counterparts, these results suggest that PIN1 could be abundant and affecting auxin transport within the polar auxin transport stream of leafy spurge plants in response to glyphosate, in a manner similar to that previously reported [32].

The increased abundance of bioactive cytokinins (Fig. 4) in the aerial shoots derived from crown buds of foliar glyphosate-treated plants is consistent with axillary bud outgrowth (Fig. 1). Ironically, although bioactive auxin levels were increased, auxin’s regulatory role on cytokinin levels and acropetal movement appears to be negated in glyphosate-treated plants. Possible explanations for the disparity of the auxin-cytokinin interaction include the local biosynthesis of cytokinin in aerial shoots, particularly since cytokinins are known to be synthesized in most plant tissues [37]. Indeed, the increased abundance of transcripts (Table 2) homologous to Arabidopsis genes involved in cytokinin biosynthesis (*AK2*, *APT2*, *APT4*, *IPT3*, *LOG5*), and decreased abundance of transcripts involved in cytokinin metabolism (*CKX3*, *UGT73C1*, *UGT74E2*) in subsequent generations of aerial shoots in response to glyphosate is consistent with the abundance of cytokinins (Fig. 4). Additionally, cytokinins are also known to impact polar auxin transport through modulation of auxin efflux carrier activity [37]. Interestingly, numerous transcripts homologous to Arabidopsis genes involved in auxin transport had a significant increase in abundance (*ABCB19*, *LAX2*, *LAX3*, *PID*, *PIN1*; Table 1). Some of these auxin transporter family members (e.g., *PINs*, and *ABC TRANSPORTERS*) also had significant changes in transcript abundance in crown buds of foliar glyphosate-treated plants prior to growth induction by decapitation [28].

Interestingly, sugar signaling, not auxin, has been identified as the main regulator of initial axillary bud outgrowth in pea [60]. Although it is difficult to determine if a similar mechanism involving sugar signaling impacts axillary bud outgrowth in aerial shoots of leafy spurge in response to glyphosate-treatment, Gene Set Enrichment Analyses (GSEA, Additional file 7) did identify both decreased and increased abundance of transcripts associated with carbohydrate metabolism and transport. Although limiting sugars through translocation to the shoot tips of axillary buds is proposed to maintain apical dominance, long-term signaling involving auxin, cytokinin and strigolactone are still thought to play a significant role in branching [60].

### Glyphosate induces transcript abundance of large gene families

Large gene families associated with detoxification processes and resistance to multiple herbicides [72–74] had significant changes in transcript abundance in aerial shoots derived from crown buds of glyphosate-treated plants. For example, members of the *P450s* (46 of 95 increased), *UGTs* (13 of 51 increased), *GSTs* (6 of 10 increased), and *ABC TRANSPORTERS* (8 of 15 increased) had significant changes in abundance (see Additional file 2). In other plant systems, P450s together with GSTs and UGTs are involved in herbicide biochemical modification through metabolism, whereas ABC TRANSPORTERS are involved in compartmentalizing herbicides and their metabolites; this four-step process is associated with herbicide resistance in some weeds [24, 72, 75-78]. As previously discussed, several homologs of Arabidopsis *P450s*, *UGTs* and *ABC TRASPORTER*s differentially expressed in this study are linked to hormone biosynthesis or signaling (Tables 1–5), and it is possible that other members of these families (see Additional file 2) could be playing some role in reducing the phytotoxic effects of residual glyphosate. Assuming these leafy spurge homologs perform similar functions, significant changes in abundance of these gene families suggest that detoxification processes could play some direct or indirect role in reducing the phytotoxic effects of glyphosate. Alternatively, because glyphosate can rapidly disrupt plastids, chloroplasts, and the endoplasmic reticulum in plants [79, 80], and some leafy spurge homologs of P450s (see Additional file 2) are known to be localized in these organelles in other systems [81], it is also possible that residual glyphosate impacts cellular organelles in UABs and could help explain the differential abundance of P450s observed in this study.

### Plants treated with glyphosate have reduced levels of bioactive GA

Even though bioactive GAs were not detectable in any samples (Fig. 5), the results indicate that precursors of bioactive GAs were increased, but the abundance of bioactive GAs may be reduced in subsequent generation of aerial tissues derived from crown buds of glyphosate-treated plants. Further, the decreased abundance of a transcript (*GA3OX1*) involved in synthesis of bioactive GAs and increased abundance of transcripts (*GA2OX2*, *GA2OX4*) involved in catabolism of bioactive GAs (Table 3, and Fig. 5), support the decreased abundance of bioactive GAs observed in glyphosate-treated plants. Overall, these results indicate that downstream GA biosynthesis and signaling are negatively impacted, which could contribute to inhibition of stem elongation of tissues derived from crown buds of foliar glyphosate-treated leafy spurge. This hypothesis is supported by the fact that certain commercial chemicals used to stunt growth (e.g., phosphon D, cycocel, ancymidol and paclobutrazol) do so by inhibiting GA biosynthesis [82].

## Conclusions

Translocation of foliar applied glyphosate and its sequestration into the root system of leafy spurge [12] caused irreversible effects to the main SAM of UABs prior to growth-induction by decapitation of the directly treated aerial tissues. This theory is supported by the increased abundance of shikimate in crown buds post foliar glyphosate-treatment and prior to growth-induction (Fig. 2). After growth-induction by decapitation, this irreversible effect, in turn, causes the main stem of subsequent generations of aerial tissue to lose apical dominance, thus impacting axillary bud outgrowth. Alternatively, the potential remobilization of root-sequestered glyphosate would be consistent with apical meristems of growing shoots acting as sink tissues [15, 83], which is supported by the fact that SNEA (see Additional file 4) highlighted numerous molecular processes associated with SAM maintenance containing transcripts with increased abundance (6 weeks post-decapitation). Collectively, these two hypotheses provide insights into potential mechanisms resulting in glyphosate-induced phenotypes that have reduced main stem elongation and increased branching due to axillary bud outgrowth (Fig. 1).

The results of this study are consistent with the known involvement of phytohormone cross-talk to regulate branching. The increased abundance of bioactive auxin and cytokinin (Fig. 2) and *PIN1* (Table 1) in subsequent generations of aerial shoots (6 weeks post-decapitation) in response to glyphosate-treatment is consistent with axillary bud outgrowth, assuming auxin is being exported from buds into the polar auxin transport stream [70, 71]. Additionally, the increased abundance of cytokinins in subsequent generations of aerial tissues derived from UABs of glyphosate-treated plants would also be consistent with the increased abundance of transcripts involved in cytokinin biosynthesis (Fig. 4) or associated with cell division processes (Table 2). The increased abundance for homologs of *MAX1* and *MAX3* suggest that strigolactone biosynthesis may be activated in response to the residual effects of glyphosate. However, in our system, the increased abundance of a *PIN1* homolog and branching in response to glyphosate are counterintuitive to increased strigolactone signaling, which can inhibit branching through regulation of auxin transporters such as PIN1 [31, 32, 69].

The differential abundance of transcripts for *P450s*, known to be localized in plastids, endoplasmic reticulum, and mitochondria, support glyphosate’s impact on disruption of these intracellular organelles, as demonstrated in other plant systems [79, 80]. Further, the differential expression of numerous *P450s*, *GSTs*, *UGTs*, and *ABC TRANSPORTERS* in subsequent generations of aerial shoots in response to glyphosate (Additional file 2) could suggest the occurrence of detoxification processes through metabolism and/or compartmentalization in these tissues. The fact that homologs of *ABC TRANSPORTERS*, *PIN*s, and *PILS* also had differential abundance in crown buds of leafy spurge in response to glyphosate [28] add further support for molecular processes associated with membrane transport and/or sequestration being impacted by glyphosate-treatment prior to growth-inducing decapitation. Although further research will be needed to determine the exact mechanism leading to these glyphosate-induced phenotypes, this research provides insights for molecular processes associated with stunted growth and increased branching from underground adventitious buds of leafy spurge.

## Material and Methods

### Plant material, glyphosate-treatments, and vegetative growth

Leafy spurge plants were propagated from a uniform biotype (1984-ND001) in cone-tainers and maintained in a greenhouse as described by Anderson and Davis [84]. After conducting dose–response experiments using concentration of 0 to 6.72 kg ha^−1^ glyphosate as active ingredient, aerial tissues of four-month old greenhouse grown leafy spurge plants were treated with 0 or 2.24 kg ha^−1^ glyphosate plus 0.25 % surfactant as described by Doğramacı et al. [28]. Plants were returned to growth-conducive conditions in a greenhouse after foliar glyphosate-treatment. Each experiment included four biological replicates; aerial tissues of eight plants from each replicate were decapitated 7 days post-application to determine the vegetative growth rate from crown buds as previously described by Foley et al. [85]. After six weeks of vegetative growth from crown buds of foliar glyphosate-treated and control plants, shoot heights were measured and analyzed using the generalized linear mixed model (PROC GLIMMIX) procedure of SAS 9.2. Further, these same cone-tainers were used to determine the longevity of glyphosate’s effects on vegetative growth from UABs of glyphosate-treated and control plants; every six weeks aboveground shoots were decapitated for three additional re-growth cycle. However, only the first generation of aerial tissues derived from UABs of glyphosate-treated and control plants were harvested and flash frozen in liquid nitrogen and stored in −80 °C for further experiments i.e., shikimate and phytohormone quantification, and transcript analyses by RNAseq and qRT-PCR. Additionally, aerial tissues of leafy spurge plants, which were directly exposed to the treatments and their crown buds were collected for shikimate quantification 7 days after the treatments.

### Quantification of shikimate levels

Quantification of shikimate in aerial tissues directly exposed to the treatments (7 days post-treatment), crown buds of foliar treated plants (7 days post-treatment), and aerial tissues derived from crown buds of foliar treated plants (six weeks after growth-inducing decapitation) were accomplished spectrophotometrically [45, 86]. In brief, 0.5 gram of frozen tissue was extracted in 2 mL 0.25 N HCl at room temperature. 250 μL of this extract was added into one ml 0.25 % (w/v) periodic acid/0.25 % (w/v) meta-periodate and incubated at 37 °C for 15 minutes. 1 mL of 0.6 M NaOH/0.22 M Na_2_SO_3_ was added into the sample and read by a spectrophotometer (Beckman, DU 7400); 510 μM shikimate (Sigma) was used as the standard. These assays included four biological and two technical replicates; technical replicates were averaged prior to calculating mean of biological replicates and standard error.

### Hormone profiling

Quantification of phytohormones (ABA and ABA metabolites, auxins, cytokinins, and gibberellins) in the eight aerial tissue samples (2 treatments [controls and 2.24 kg ha^−1^ glyphosate] × 4 biological replicates) derived from crown buds of foliar glyphosate-treated plants was conducted at the NRCC-PBI (Saskatoon, SK, Canada) by UPLC-ESI-MS/MS. Deuterated forms of each of the hormones were used as internal standards [87, 88]. Extraction and purification of samples and quantification of phytohormones was performed following a procedure described in Chiwocha et al. [89, 90]. Profiling data obtained from four biological replicates were used to calculate the mean and standard error. The list and abbreviations of hormone profiles are provided in Additional file 5.

### RNA extraction, cDNA library preparation, and transcript analyses by RNAseq

Total RNA was purified from the eight aerial tissue samples derived from crown buds of foliar glyphosate-treated plants according to the pine tree RNA extraction protocol [91]. The RNA was treated with amplification grade DNase1 (Invitrogen), quantified by the Qubit® 2.0 Fluorometer, and quality was confirmed by agarose gel electrophoresis. The RNAseq libraries were prepared with the TruSeq Stranded mRNA LT Sample Preparation Kit (Illumina, Cat. N° RS–122–2101) starting from 1 μg of total RNA. The pool of barcoded RNAseq libraries was quantified by qRT-PCR using the Library Quantification kit (Kapa Biosystems, Cat. N° KK4824). The size range of the final cDNA libraries was determined on an Agilent bioanalyzer DNA7500 DNA chip (Agilent Technologies). The cDNA libraries were sequenced on one lane for 151 cycles from each end of the cDNA fragments on a HiSeq2500 using a TruSeq SBS sequencing kit version 1 (Illumina). The sequence images were transformed with the Real Time Analysis 1.17.21.2 Illumina software to bcl files, which were demultiplexed to fastq files with CASAVA version 1.8.2. The quality-scores line in fastq files processed with Casava1.8.2 use an ASCII offset of 33 known as Sanger scores.

### Transcriptome assembly, annotation and expression analyses

The Trinity package version trinityrnaseq-r2013-02-25 [92] was utilized to assemble the transcriptome from the RNASeq data. Prior to assembly, adaptor sequences were trimmed and so were any low quality bases (<30 phred score) from both ends of the sequence. Sequences of 50 nucleotide and higher were utilized for the Trinity assembly. The assembly was performed as per guidelines in the published protocols [92], and on the Trinity website (http://trinityrnaseq.sourceforge.net/#running_trinity). The abundance of every gene (component) in each of the eight samples was determined by performing alignment and abundance estimation using the RSEM package [93] (version 1.2.3). Only those genes that had at least one sample with the transcripts per million (TPM) value of ≥ 0.5 were used as input for the next step. Differential gene expression was performed at the level of genes with the EBSeq package [94] (version 1.0) using the median normalization approach and 10 iterations of the algorithm used by EBSeq. Genes were called differentially expressed if they had an FDR value of ≤0.05.

Annotation of the assembled transcriptome and predicted proteome was performed with the Trinotate package (version trinotate_r20130826). Transdecoder, a Perl script packaged with Trinity, was used to predict proteins, yielding the most likely longest-ORF peptide candidates file best_candidates.eclipsed_orfs_removed.pep. Analyses were performed as detailed in the Trinotate manual (http://trinotate.sourceforge.net/), with the following noted exceptions. BLASTx of assembled transcripts and BLASTp most likely longest-ORF peptide candidates against UniProt-SwissProt database were run with the options “-evalue 10E-3 –max_target_seqs 5” [95]. For more reliable functional annotation, BLASTx of Trinity.fasta and BLASTp of best_candidates.eclipsed_orfs_removed.pep against Arabidopsis peptides were performed (Athaliana_167_protein.fa from Phytozome v9.0). BLAST output files were parsed to 1 top hit per query with an in-house Perl script prior to insertion in the Trinotate SQLite database (http://trinotate.sourceforge.net/#LoadingSQLite). After extraction of the Trinotate annotation report from the database (http://trinotate.sourceforge.net/#OutputReport), Arabidopsis annotations were added using an in-house Perl script. The expression data is deposited at Gene Expression Omnibus (http://www.ncbi.nlm.nih.gov/geo/) as GEO dataset query GSE56509.

### qRT-PCR analyses

qRT-PCR was utilized to confirm transcript profiles obtained from RNAseq for selected genes. Sequences from a leafy spurge EST database [96] were used for designing primer pairs using the Primer-Select of Lasergene 8 software program (DNASTAR, Inc., Madison, WI). These primer pairs were used to quantify abundance of selected transcripts (see Additional file 3). Total RNA samples were reverse transcribed into complementary DNA (cDNA) as described in Doğramacı et al. [28]. In brief, 5 μg of total RNA was treated with DNase1 amplification grade (Invitrogen), and reverse transcribed using Super Script First-Strand Synthesis Kit3 (Invitrogen) in a 20 μl volume according to the manufacturer’s instructions. After cDNA synthesis, each reaction was diluted to 800 μl total volume and stored at −80 °C. For qRT-PCR reactions, 1 μl total cDNA was added to a 10 μl PCR reaction mixture containing 5 μl of LightCycler® 480 SYBR Green I Master and 0.5 μl of each primer-set. Transcript abundance was measured from three technical and four biological replicates using a LightCycler 480 II (Roche). All transcript values were normalized using the reference genes ARF2, PTB, SAND, and ORE9 identified by Chao et al. [97]. QbasePLUS version 2.4 software (Biogazelle, Ghent, Belgium) was used to normalize expression values. Values from four biological replicates were averaged and data from control samples (0 kg ha^−1^) were used for baseline expression. Gene abbreviations and descriptions of all putative homologous leafy spurge genes included throughout this report were obtained from an Arabidopsis website (www.arabidopsis.org) and are presented in (see Additional file 2). Pathways in Figs. 2–6 were developed based on the PlantCyc website and/or available literature on hormone biosynthesis/signaling, and more details can be found at http://pmn.plantcyc.org/PLANT/class-tree?object=Pathways.

### SNEA and GSEA of transcriptome data

The entire transcriptome dataset (i.e., transcripts per million values; see Additional file 2) was analyzed using Ariadne Pathway Studio 9.0 Software-Resnet Plant Version 2.1 (Ariadne Genomics Inc., Rockville, MD, USA) to obtain SNEA and GSEA using Mann–Whitney U test algorithm with a 0.05 enrichment p value cutoff. Central hubs of sub-networks were obtained by SNEA (Additional file 4) using the customized advanced parameters to identify the most important biological processes, transcription factors, binding partners, miRNA targets, and protein modification targets for the transcriptome data. GSEA was used to identify predefined sets of genes over-represented between treatments (Additional file 7) based on gene ontology (http://www.geneontology.org/) or AraCyc metabolic pathways (http://pmn.plantcyc.org/).

## Additional files

Additional file 1:
**Vegetative growth from underground adventitious buds of glyphosate-treated and control plants.** Aerial tissues of control and glyphosate-treated (0 or 2.24 kg ha^−1^) leafy spurge plants were decapitated to induce the first subsequent generation of aerial shoots from crown buds. Then, three additional decapitation and vegetative growth (VG-2, −3, −4) measurements were repeated every six weeks to monitor longevity of glyphosate’s effects on vegetative growth. Vertical bars indicate 95 % confidence limits. Additional file 2:
**RNAseq results: List of selected genes; list of 9,283 differentially expressed transcripts with Arabidopsis annotation; list of 12,918 differentially expressed transcripts; raw transcripts per million (TPM) values for each treatment and biological replicate.**
Additional file 3:
**List of primer sequences used for qRT-PCR assays, and correlation coefficient values for qRT-PCR vs. RNAseq.**
Additional file 4:
**Sub-network enrichment analyses.**
Additional file 5:
**List of phytohormone profiles performed.**
Additional file 6:
**Ethylene biosynthesis and signaling pathway.**
Additional file 7:
**Gene set enrichment analyses.**
