# Supplementary material for: Glyphosate’s impact on vegetative growth in leafy spurge identifies molecular processes and hormone cross-talk associated with increased branching
Source: BMC Genomics. 2015 May 19;16(1):395. doi: 10.1186/s12864-015-1627-9 (PMC4437557; doi:10.1186/s12864-015-1627-9)

Additional file 1. Additional decapitation and vegetative growth from underground adventitious buds of glyphosate treated and control plants to monitor longevity of glyphosate's effects on vegetative growth. Aerial tissues derived from crown buds of control and glyphosate treated (0 or 2.24 kg/ha) leafy spurge plants were initially decapitated 7-days after the treatment (see Figure 1). Three subsequent decapitation and vegetative growth (VG-2, -3, -4) measurements were repeated every six weeks; shoot heights were measured prior to decapitation. Vertical bars indicate 95% confidence limits.

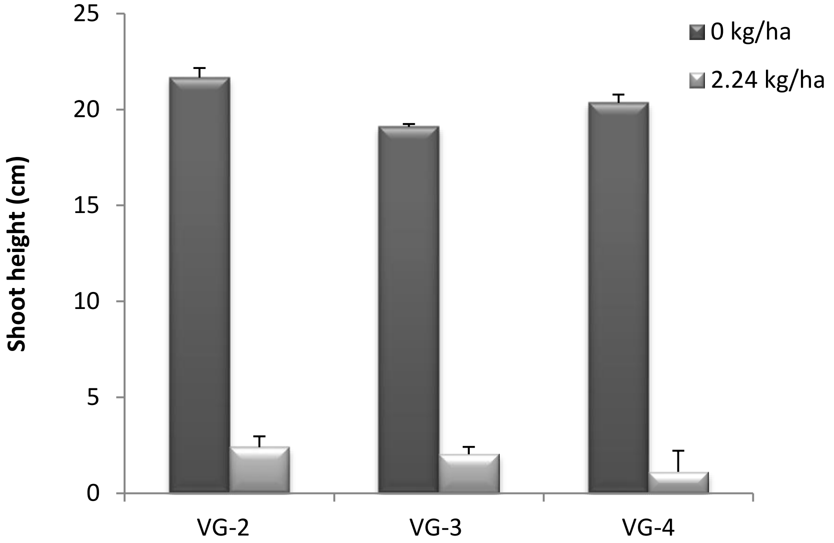

Supplement: Additional file 1: — Vegetative growth from underground adventitious buds of glyphosate-treated and control plants. Aerial tissues of control and glyphosate-treated (0 or 2.24 kg ha−1) leafy spurge plants were decapitated to induce the first subsequent generation of aerial shoots from crown buds. Then, three additional decapitation and vegetative growth (VG-2, −3, −4) measurements were repeated every six weeks to monitor longevity of glyphosate’s effects on vegetative growth. Vertical bars indicate 95 % confidence limits. [file 12864_2015_1627_MOESM1_ESM.pdf]
