# Supplementary material for: Glyphosate’s impact on vegetative growth in leafy spurge identifies molecular processes and hormone cross-talk associated with increased branching
Source: BMC Genomics. 2015 May 19;16(1):395. doi: 10.1186/s12864-015-1627-9 (PMC4437557; doi:10.1186/s12864-015-1627-9)
Supplement: Additional file 5: — List of phytohormone profiles performed. [file 12864_2015_1627_MOESM5_ESM.pdf]

Additional file 5. Phytohormone profiles monitored in aerial tissues derived from crown buds of foliar glyphosate-treated (0 or 2.24 kg ha^{-1}) leafy spurge plants.

**ABA & metabolites**		
	ABA	*cis*-Abscisic acid
	ABAGE	Abscisic acid glucose ester
	DPA	Dihydrophaseic acid
	PA	Phaseic acid
	7'OH-ABA	7'-Hydroxy-abscisic acid
	neo-PA	*neo*-Phaseic acid
	t-ABA	*trans*-Abscisic acid
**Auxins**		
	IAA	Indole-3-acetic acid
	IAA-Asp	N-(Indole-3-yl-acetyl)-aspartic acid
	IAA-Glu	N-(Indole-3-yl-acetyl)-glutamic acid
	IAA-Ala	N-(Indole-3-yl-acetyl)-alanine
	IAA-Leu	N-(Indole-3-yl-acetyl)-leucine
	IBA	Indole-3-butyric acid
**Cytokinins**		
	t-ZOG	(*trans*) Zeatin-O-glucoside
	c-ZOG	(*cis*) Zeatin-O-glucoside
	t-Z	(*trans*) Zeatin
	c-Z	(*cis*) Zeatin
	dhZ	Dihydrozeatin
	t-ZR	(*trans*) Zeatin riboside
	c-ZR	(*cis*) Zeatin riboside
	dhZR	Dihydrozeatin riboside
	iP	Isopentenyladenine
	iPR	Isopentenyladenosine
**Gibberellins**		
	GA1	Gibberellin 1
	GA3	Gibberellin 3
	GA4	Gibberellin 4
	GA7	Gibberellin 7
	GA8	Gibberellin 8
	GA9	Gibberellin 9
	GA19	Gibberellin 19
	GA20	Gibberellin 20
	GA24	Gibberellin 24
	GA29	Gibberellin 29
	GA34	Gibberellin 34
	GA44	Gibberellin 44
	GA51	Gibberellin 51
	GA53	Gibberellin 53
