# Supplementary material for: Glyphosate’s impact on vegetative growth in leafy spurge identifies molecular processes and hormone cross-talk associated with increased branching
Source: BMC Genomics. 2015 May 19;16(1):395. doi: 10.1186/s12864-015-1627-9 (PMC4437557; doi:10.1186/s12864-015-1627-9)

**Additional file 6.** Simplified pathway of ethylene biosynthesis and signaling in plants. Ethylene is produced from L-methionine; methionine is used to form S-adenosylmethionine (SAM), followed by conversion of SAM to 1-aminocyclopropane-1-carboxylic acid (ACC), which is the immediate precursor of ethylene. Red italic text indicates the genes involved in particular step/s of the ethylene biosynthesis pathway. The ethylene signaling pathway involves five ethylene receptors (ETR1, ETR2, EIN4, ERS1 and ERS2), the MAPKKK-like protein CTR1, EIN2, and the EIN3 and ERF transcription factor families.

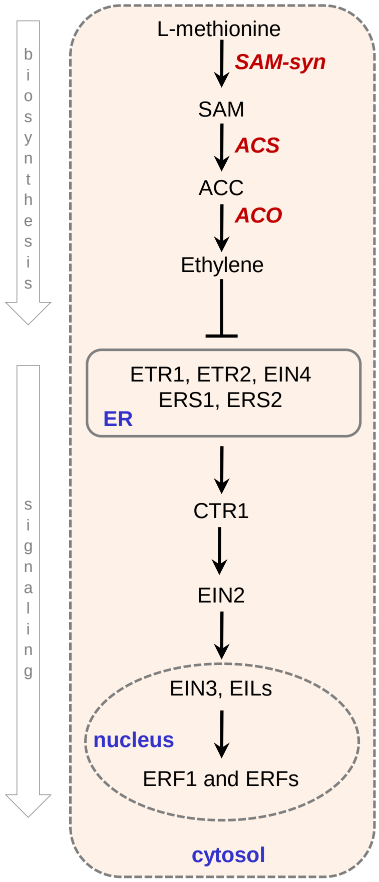

Supplement: Additional file 6: — Ethylene biosynthesis and signaling pathway. [file 12864_2015_1627_MOESM6_ESM.pdf]
